# Associations Between Psychopathological Symptom Severity Amid the Pandemic and the Childhood Sociodemographic Environment

**DOI:** 10.7759/cureus.56458

**Published:** 2024-03-19

**Authors:** Dean M Pucciarelli, Rahul Ramasubramani, Charles H Trautmann

**Affiliations:** 1 Psychiatry, Rutgers Robert Wood Johnson Medical School, Piscataway, USA; 2 Psychiatry, Rutgers Robert Wood Johnson Medical School, New Brunswick, USA; 3 Psychology, Cornell University, Ithaca, USA

**Keywords:** anxiety, depression, childhood, social class, socioeconomic status, pandemic, covid-19

## Abstract

It is well-documented that childhood socioeconomic status (SES) is associated with various health conditions in adulthood. Here, we examine the extent to which childhood SES is associated with COVID-19 pandemic anxiety and depression. Participants (n = 212), recruited from Amazon Mechanical Turk, were assessed for depression and anxiety in February 2022 for both the current context and retrospective self-perceived early pandemic depression and anxiety (April 2020). Participants also reported childhood SES and current demographics. Consistent with predated findings, we show a strong, positive correlation between depression and anxiety under both conditions. Paternal unemployment in childhood was associated with increased anxiety, while maternal occupation was not. High household education in childhood was generally associated with greater anxiety and depression, similar to past studies examining education levels and depression. However, the shift from high school to post-secondary degrees (trade school and associate’s) was associated with decreased anxiety and depression, which may reflect “essential work” careers, therefore indicating a dualism. Growing up in crowded, de-individualized spaces was associated with lower anxiety and depression, suggesting better conditioning for the imposition of COVID-19 quarantines. Pandemic-related unemployment was associated with an increase in anxiety and depression. Strong political views, regardless of ideology, were associated with increased anxiety. Finally, participants in our cohort perceived their mental health to be worse in the early pandemic for anxiety and depression, up 6.6% and 7.9%, respectively. Our work suggests a complex relationship between SES, demographics, and anxiety and depression during the pandemic. These findings emphasize the importance of exploring the dynamics between early SES and mental health in adulthood, particularly during extended societal stressors.

## Introduction

Growing evidence reveals that the development of adult health conditions may be impacted by childhood socioeconomic status (SES), supporting a life-course approach to well-being [1]. In a meta-analysis, Spencer et al. discussed the association between physical health disparities and childhood SES, finding that lower SES in childhood typically corresponds to higher rates of health challenges in adulthood [2]. This trend is also evident in studies of mental health, where lower childhood SES is associated with poorer mental health outcomes in adulthood [3-5]. Several studies have documented the association between parental social class factors, such as income, unemployment, and level of education, with the severity of anxiety and depression in adulthood [3,6,7]. Many have also reported an association between physical environment SES factors in childhood, such as home overcrowding and access to hot water, with negative mental health outcomes in adulthood [8,9]. Because physical health not only correlates with, but can directly impact, mental health, it is therefore also important to examine the impact of the physical environment on mental health, especially amid lasting societal stressors such as the COVID-19 pandemic [10,11]. For example, it has been long known that home overcrowding can have abundant and significant mental health consequences [8,9].

There is also evidence that several demographic factors, such as political leader preferences and current unemployment, may be linked to childhood SES and adverse experiences [12-14]. On some occasions, these demographic factors are also linked with psychopathology; for example, it has been well-established that unemployment may trigger the onset of anxiety and depressive disorders [15,16]. While links between unemployment and psychopathology present with more clear mechanisms, associations between political views and psychopathology may be dependent on the current socio-political environment, making the relationship more complex. For example, levels of depression and anxiety may have been impacted by masking mandates implemented during the COVID-19 pandemic [17].

Even though these significant changes in mental health over a short period of time have been found to occur across individuals regardless of nationality or demographic groups, the pandemic has also brought to light the severity of health disparities among marginalized groups. Individuals of lower SES have had considerably worse health outcomes amid the COVID-19 pandemic; the largest rate of sickness and death from the SARS-CoV-2 virus comes from individuals of lower SES [18]. Similarly, a high incidence of anxiety and depression has been found among people with lower SES amid the pandemic in the United Kingdom (UK) [19]. While these associations are established, the relationship between childhood SES and adult anxiety and depression during the pandemic has yet to be thoroughly examined. This relationship is especially important to investigate, given that childhood SES is often correlated with adult SES, and adult SES has been found to correlate with mental health amid the pandemic [20,21].

The COVID-19 pandemic has been consistently linked with a societal upsurge in depression and anxiety [22-25]. Pairing this knowledge with evidence of pandemic-related physical health disparities (i.e., increased COVID-19 mortality in lower SES individuals), it is essential to examine the manifestation of related psychopathology among lower SES individuals. One of the ways that lower SES has been associated with pandemic-related anxiety is through personal economic situations; several studies have examined the severe impact of the pandemic on mental health in lower-income and food-insecure communities [26,27]. However, individuals with low SES are not the only ones with pandemic-related mental health consequences. There is some indication that people with higher SES experienced negative mental health impacts, such as during the economic challenges that resulted from collapsed stock and real estate markets during the early COVID-19 pandemic [28,29]. Therefore, it is important to assess possible mechanisms by which anxiety and depression amid the pandemic relate to various SES factors and demographics.

In addition to better understanding the impact of pandemic-related stressors on mental health, it is also important to consider habituation to stressors, and possible mitigating factors, such as vaccine availability, on psychopathology during the COVID-19 pandemic. Studies have documented a variety of coping strategies used by individuals to manage stress produced by the COVID-19 pandemic at home and in the workplace [30,31]. It is therefore possible that habituation to ongoing pandemic-related stress may play a role in a reduction of anxiety and depression; as coping strategies become utilized over the course of the pandemic, a resulting drop in anxiety and depression symptoms may follow. Furthermore, it has been suggested that the vaccination rollout may have reduced worry about health in children amid the COVID-19 pandemic, which may have had an impact on psychopathologies such as anxiety [32]. It is thus important to assess the stages of the COVID-19 pandemic and possible changes in anxiety and depression symptom severity as the pandemic progresses.

Purpose of the present study

In light of health disparities amid the COVID-19 pandemic and increased rates of psychopathology such as anxiety and depression, it is important to investigate how childhood SES may be associated with the severity of mental health symptoms during the pandemic. In particular, socioeconomic class and physical environment surroundings in childhood may relate to differential predispositions to pandemic-related psychopathology. In the current study, we examined a series of childhood SES, current SES, and demographic variables in relation to early- and late-stage pandemic depression and anxiety symptoms. Consistent with past research on adult health outcomes and childhood SES, we hypothesized that lower childhood SES and associated current demographics would relate to higher levels of depression and anxiety in both the early and late stages of the COVID-19 pandemic. 

This article was previously presented as a meeting abstract at the 2022 Cornell University Undergraduate Psychology Conference, on May 14, 2022, and has been accepted as a meeting abstract at the 2024 Rutgers New Jersey Medical School Health Systems Science Conference to be held on April 8, 2024.

## Materials and methods

Participants

Survey participants were recruited using the Amazon Mechanical Turk website. Demographics were assessed on the basis of age, sex, political views, pandemic-related unemployment and unemployment risk (participants were able to indicate if their employment type was at risk for termination, defined as being deemed under non-essential work as a result of the pandemic, even if they were not terminated), and COVID-19 vaccination status. A total of 299 participants completed the study, of these, 87 participants were omitted, resulting in a total of 212 analyzed individuals. Participants were omitted for failing one or more of five attention checks. The age range of the study participants was 18-76 years, with an average of 36 and a median of 33 (SD = 12.40). Additionally, 49.5% of participants were assigned a birth sex of male, and 50.5% of individuals were assigned female; we show a roughly equal distribution of participants by biological sex (Appendix B, Table 5). Our measured sample was predominantly White with 85.4% of individuals as White, 8.5% Black or African American, 2.4% Asian, 2.8% mixed races, and 0.9% not indicated. In terms of ethnicity, 18.4% of participants identified as Hispanic or Latino, 78.8% did not, and 2.8% did not indicate ethnicity. Ninety-one percent of participants received at least one dose of vaccine against COVID-19, 5.5% did not, and 2.5% did not indicate their vaccination status. Meanwhile, 44.8% of participants claimed to be unemployed or at risk for unemployment as a result of the pandemic, 48.6% of participants did not, 2.4% did not answer, and 4.3% were unsure. The average political views for participants on a scale from 1 (strong liberal) to 5 (strong conservative) were 2.81 (SD = 1.47), indicating a neutral sample on average with a slight liberal skew.

Measures and procedure

An online survey was administered via Qualtrics (Silver Lake, Seattle, Washington, DC) to currently and retrospectively assess depression and anxiety symptom severity in the early and late pandemic (our time point of current symptom measurement) in addition to the aforementioned demographic variables. The survey consisted of a consent form, the Center for Epidemiological Studies Depression Scale (CESD-10), the General Anxiety Disorder Scale (GAD-7), and demographic questions [33,34]. Consenting participants were not required to answer questions regarding sex, race, ethnicity, or vaccination status. Individuals were provided an “unsure” option for questions on childhood access to heating and hot water. For current pandemic-related unemployment risk, participants were able to select “unemployed with no current intent to find work” and an “unsure” option. Classifications for social class by parental occupation were used according to the Registrar General’s social classifications, with participant careers and groupings (provided in Appendix B, Table 7) [35]. Participants were asked to report questions about childhood SES when they were eight years of age, or “middle childhood,” a time at which childhood development has been suggested to be most influenced by social context [36]. The survey did not have a minimum or maximum time limit and was open for six days between February 15 and February 20, 2022. Surveys were posted daily on Amazon Mechanical Turk between 8:00 AM and 8:00 PM Eastern Standard Time. The mean time spent on the survey was 8.01 minutes (SD = 5.36). Participants were not told that survey questions measured clinical symptomatology. We limited our requested “hits” on Amazon Mechanical Turk to 50 per day, on average, to ensure that all responses were filled during daylight hours. Similarly, to prevent errors in situational changes due to the constantly changing nature of the pandemic, we recorded all measurements within six days, resulting in a total sample size of 299. Limited statistical analyses were employed to mitigate the possibility of data distortion in our findings; specifically, we performed a paired sample t-test, independent samples t-tests, Pearson correlations, and analyses of variance (ANOVAs) with post-hoc Tukey tests using Statistical Product and Service Solutions (SPSS, version 26; IBM SPSS Statistics for Windows, Armonk, NY). ANOVA was chosen instead of regression analysis due to the absence of interval data; our reported variables do not have specified intervals. For example, the magnitude of difference between the Registrar General's social class, household education, or employment levels cannot be discretely defined.

Measurement of anxiety and depression

The GAD-7 was used to measure clinical symptoms and severity of anxiety. The outcome of this scale is determined using a total score of items; outcomes are scored as minimal anxiety (0-4), mild anxiety (5-9), moderate anxiety (10-14), and severe anxiety (15-21). The CESD-10 was used to measure clinical symptoms and severity of depression; depression intensity on this scale is determined by a total score on a gradient of 0-30, rather than a rigid grouping of scores by categories. Current and perceived early pandemic depression and anxiety symptom levels were retrospectively measured in a recall-based, early pandemic condition before the release of a COVID-19 vaccine (specified as April 2020 in our survey), and, in the current context (February 2022, the survey date), at which time COVID-19 vaccines had been widely available for 22 months. Perceived early pandemic depression and anxiety symptoms were assessed to reflect participants’ self-understanding of their mental health in the early stages of the pandemic, as early adverse mental health has been correlated with later poor mental health outcomes [37-39]. Although the GAD-7 and CESD-10 were originally developed to record levels of depression and anxiety during the last two weeks, there is no current scale that exclusively assesses depression and anxiety retrospectively. However, this study intentionally assesses the recollection, or perception, of earlier experienced anxiety and depression. Over time, studies have indicated that symptoms of depression and anxiety can be impacted by memory, making a controlled comparison important to assess [40].

Attention checks, quality control, and exclusions

Studies have found that Amazon Mechanical Turk respondents are a trustworthy source of data with fair statistical power, particularly with the inclusion of attention-check survey questions [41-43]. The need for attention checks on this platform partly stems from that Amazon Mechanical Turk is a paid platform, where participants are provided monetary benefits for responding to questionnaires. Four distinct and separate multiple-choice attention-check questions were designed into the questionnaire. A fifth attention check was administered, in which participants were asked to report parental employment using specific format guidelines. If any of these five attention checks failed, data from that participant were excluded from the study. Other exclusions from the study included unclear or inconsistent responses (i.e., indicating very high SES for some variables and very low SES for others) and time (taking the survey in very short durations, even if all attention checks were passed). Pre-existing conditions for depression and anxiety were not assessed as an exclusionary criterion, as field evidence suggests prevalent misdiagnosis and underreported symptoms partially related to external societal stigma. Therefore, exclusions of these individuals would be likely to only exclude a fraction of those with pre-existing conditions, introducing bias into the study [44-46].

Demographics and current unemployment

Several demographic questions were asked, in addition to the preceding variables. Using a five-point Likert scale, participants were asked to indicate their current political views, from strongly conservative to strongly liberal. Participants were also asked about their current pandemic-related unemployment or unemployment risk (risk for unemployment being deemed a non-essential career during the pandemic), biologically assigned sex at birth, age, race, ethnicity, and vaccination status. Vaccinated individuals were defined as those having received at least one dose of a COVID-19 vaccine. While all survey questions were required, participants were given a “prefer not to answer” option for questions regarding biologically assigned birth sex, race, ethnicity, and vaccination status.

## Results

Early- versus late-pandemic anxiety and depression scores

A paired-sample t-test was run in Data Desk 8.3 (Data Description, Inc., Ithaca, NY) to examine the difference between early versus late GAD-7 and CESD-10 scores (n = 212); a power analysis in Statistical Product and Service Solutions (SPSS, IBM SPSS Statistics for Windows, Armonk, NY) showed acceptable levels of statistical power for a paired sample t-test for both the CESD-10 (1-β = 0.88) and GAD-7 (1-β = 0.81). Results from the paired sample t-test indicated a significant difference for both depression (p = 0.0019**, df = 210) and anxiety (p = 0.005**, df = 210). Average scores for early- and late-pandemic CESD-10 were 14.09 (SD = 6.12) and 12.97 (SD = 6.48), respectively, indicating a 7.9% perceived decrease during the pandemic. Average scores for early- and late-pandemic GAD-7 were 10.10 (SD = 5.53) and 9.43 (SD = 5.54), respectively, indicating a 6.6% perceived decrease during the pandemic. Together, these findings indicate a significant decrease in depression and anxiety symptoms on average over the course of the COVID-19 pandemic.

Childhood pandemic-related anxiety and depression correlation

Pearson’s correlation was run in R (R Development Core Team, Vienna, Austria) between CESD-10 and standardized GAD-7 scores in the early and late pandemic (Appendix C, Equation 1; n = 212). In the early pandemic, a strong, positive correlation was found between CESD-10 and GAD-7 scores (r = 0.82, p < 0.0001****). Similarly, a strong, positive correlation was found between the CESD-10 and GAD-7 in the late pandemic (r = 0.85, p < 0.0001****). (Plots are provided in Appendix A, specifically Figures 4-5).

Childhood social class: paternal occupation

Based on the average participant CESD-10 and GAD-7 score differences among paternal occupation groups, we decided to run a one-way ANOVA in SPSS. Using the Registrar General’s social classifications, we grouped individuals into classes I-V based on parental employment and created a sixth group for unemployed individuals, where class I indicated the highest SES, and class V the lowest [35]. Results indicated a statistically significant ANOVA for anxiety in the early (p = 0.016*) and late (p = 0.005**) pandemic (Appendix B, Table 8). Results for depression were not significant in both the early (p = 0.30) and late (p = 0.12) pandemic. As a result, we ran Tukey post-hoc tests in both anxiety conditions. Results indicated significantly lower anxiety for individuals in paternal social class III versus individuals with unemployed fathers at eight years of age in the early (p = 0.009**) and late (p = 0.008**) pandemic; class III includes professions such as police officers, secretaries, farmers, and plumbers. Those from paternal social class III (n = 26) indicated an average GAD-7 score of 7.85 (SD = 5.42) and 6.38 (SD = 4.83) in the early and late pandemic, respectively, and CESD-10 score of 12.85 (SD = 6.91) and 11.54 (SD = 6.98) in the early and late pandemic. Individuals with unemployed fathers at 8 years of age indicated an average GAD-7 score of 14.15 (SD = 5.57) in the early pandemic and 12.77 (SD = 5.42) in the late pandemic.

**Figure 1:**
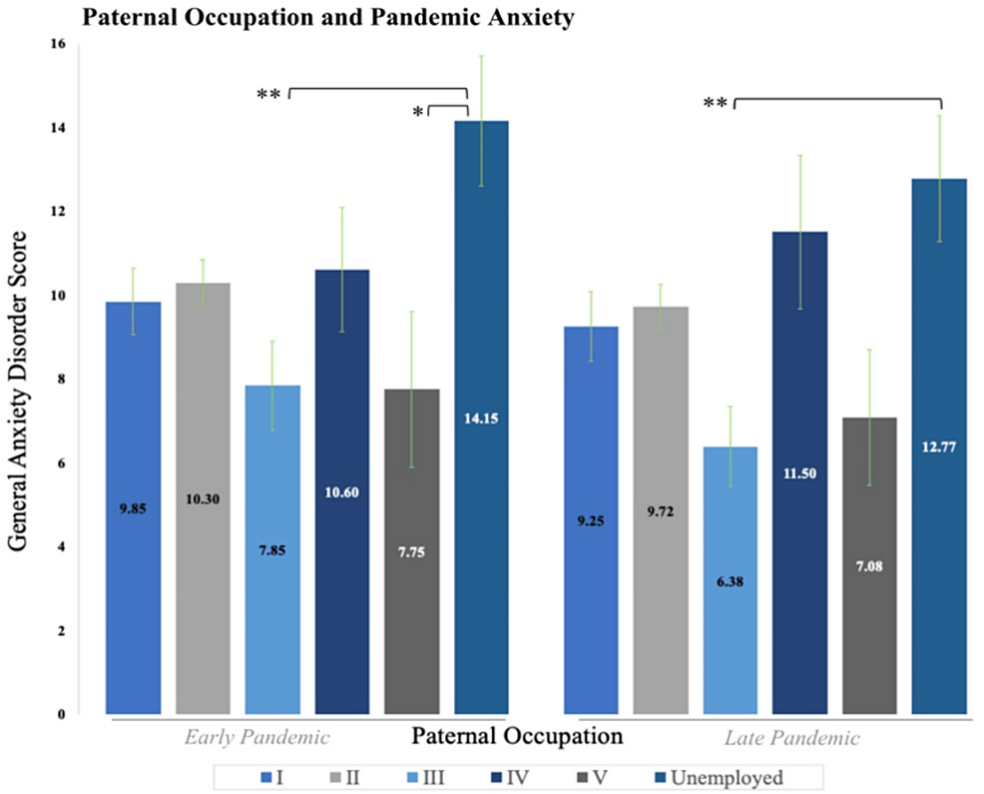
Mean GAD-7 scores for the paternal occupational class of participants when they were eight years old in the early and late pandemic. Individuals whose fathers were unemployed at eight years of age show significantly more anxiety, while those with fathers who belonged to social class III mark significantly less anxiety. p < 0.05*, p < 0.01** GAD-7: General Anxiety Disorder Scale 7

Furthermore, post-hoc tests revealed that in the early pandemic, anxiety scores for individuals with unemployed fathers were significantly higher than individuals with fathers in social class V (n = 12; p = 0.04*), but this finding was insignificant in the late pandemic (p = 0.092). Individuals with fathers in social class V at 8 years of age indicated an average GAD-7 score of 7.75 (SD = 6.40) in the early pandemic and 7.08 (SD = 5.62) in the late pandemic. While individuals in paternal social class II reported higher anxiety on average compared to social class III, the result slightly exceeded our significance level (p = 0.064) in the late pandemic. These results are indicated in Figure 1 for anxiety (and Figure 6 in Appendix B for depression).

Childhood social class: maternal occupation

The average scores across social class by maternal occupation depression and anxiety scores remained relatively constant. We ran a one-way ANOVA comparing average CESD-10 and GAD-7 scores among maternal occupation groups in the early and late pandemic. Consistent with this observation, the results from the ANOVA indicated no statistically significant differences between individuals when segmented by maternal social class at eight years of age (Appendix B, Table 9).

Childhood social class: highest level of household education

Early-pandemic anxiety and depression scores were the lowest for individuals who grew up in households where the highest academic degree was post-secondary (trade school and associates; n = 15). A one-way ANOVA indicated significance in the early (p = 0.018*) and late (p = 0.029*) pandemic for anxiety (Appendix B, Table 10). Utilizing a Tukey post-hoc test, the average scores rose significantly as education level either dropped below, or rose above, a post-secondary degree for anxiety, including high school degrees (n = 23, p = 0.015*), bachelor’s degrees (n = 132, p = 0.02*), and post-baccalaureate degrees (n = 41, p = 0.044*) in the early pandemic; this relationship only remained true compared to bachelor’s degrees in the late pandemic (p = 0.014*).

In the early pandemic, the average GAD-7 scores for individuals by highest household education were high school (11.35; SD = 6.69), post-secondary (5.93; SD = 4.46), bachelor's (10.24; SD = 5.10), and post-baccalaureate (10.24; SD = 5.86), whereas in the late pandemic, the average GAD-7 scores for post-secondary was 5.4 (SD = 4.22) and 9.87 (SD = 5.18) for bachelor's.

**Figure 2:**
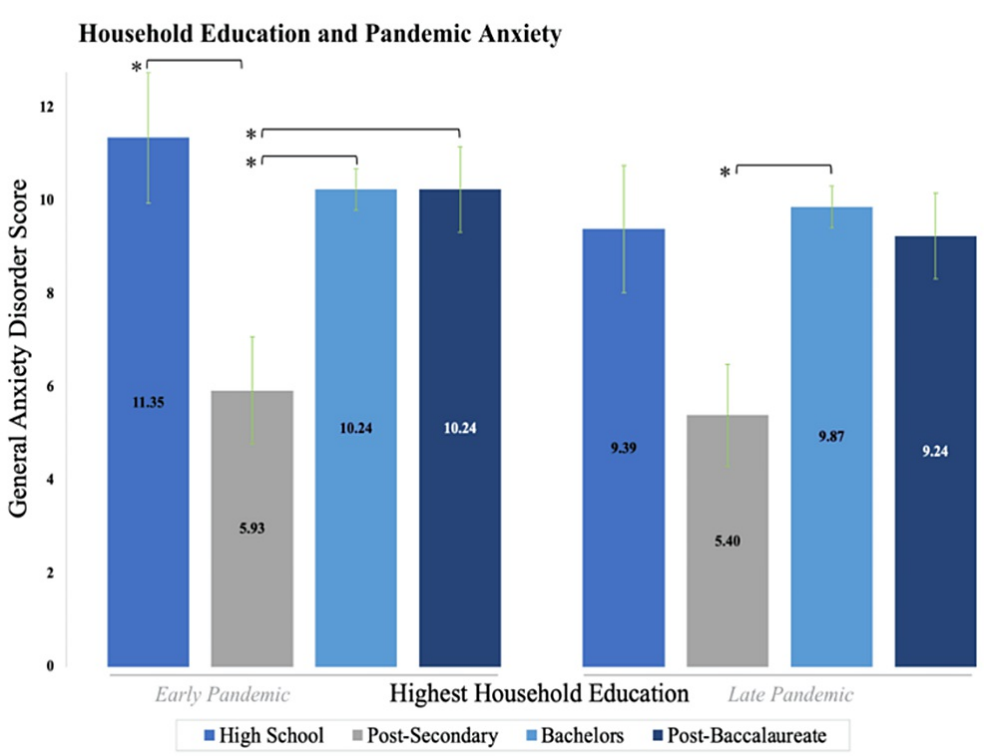
Reported mean GAD-7 scores for individuals by the highest level of education in the household at eight years of age in the early and late pandemic. Individuals whose parents completed the highest educational level of a high school, bachelor's, or post-baccalaureate reported higher levels of anxiety. Individuals whose parents had completed a post-secondary degree (trade school, associates) were associated with lower anxiety levels in both the early and late pandemic.

**Figure 3:**
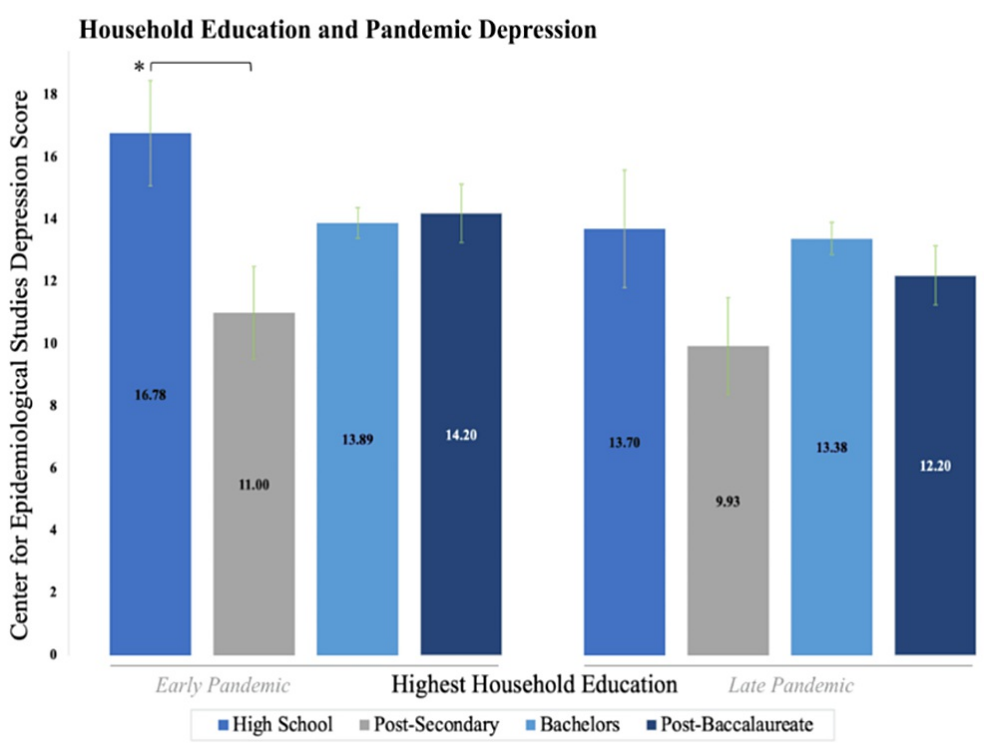
Reported mean CESD-10 scores assessed for individuals based on the highest level of education in the household at eight years of age in the early and late pandemic. Individuals whose parents had completed a post-secondary degree (trade school, associates) were associated with lower depression levels in the early pandemic as compared to parents with a high-school degree. p < 0.05* CESD-10: Center for Epidemiological Studies Depression Scale 10

ANOVA results indicated a significant relationship between education and depression in the early pandemic (p = 0.037*) but were not significant in the late (p = 0.20) pandemic. A Tukey post-hoc test of early pandemic results revealed significantly higher depression in individuals whose parents had only a high school degree compared to those whose guardians held a post-secondary degree (p = 0.022*), like our results for anxiety. The mean early-pandemic CESD-10 score for high school degrees was 16.78 (SD = 8.1) in contrast with 11 (SD = 5.73) for post-secondary degrees. Results for this portion of the study are provided in Figures 2-3.

Current social class: current unemployment 

Independent sample t-tests were run between individuals who indicated that they were unemployed or at risk for unemployment as a result of the pandemic (n = 103) and individuals who were both employed and not at risk for pandemic-related unemployment (n = 95). Results indicate that individuals who were unemployed or at risk had 20% higher anxiety in both the early (p = 0.005**) and late (p = 0.011*) pandemic compared to those employed and not at risk. Individuals who are unemployed or at risk had 18% higher depression in the late (p = 0.0047**) pandemic, but there was no significant influence of employment status on depression in the early (p = 0.18) pandemic.

In the context of early pandemic versus late pandemic reported mental health outcomes, employment status had no clinically significant influence on the general decreasing trend in anxiety from the early to late pandemic (-7.2% and -6.8% for unemployed/at-risk and employed/no-risk, respectively). However, for depression, scores decreased significantly more during the pandemic for employed people when compared to unemployed/at-risk individuals (-14.7% vs. -4.0% for employed and unemployed/at-risk, respectively). All mean scores are presented in Table 1.

**Table 1:** Pandemic unemployment or unemployment risk relations to anxiety and depression. Results suggest that individuals who were unemployed because of the pandemic, or at risk for unemployment because of the pandemic, were associated with higher levels of anxiety and depression. p < 0.05*, p < 0.01**

	n	Early Depression	σ	Late Depression	σ	Early Anxiety	σ	Late Anxiety	σ
Unemployed (or at risk)	103	14.76	5.79	14.17	6.03	11.07	5.02	10.27	5.3
Employed (or no risk)	95	13.58	6.56	11.59	6.63	8.87	5.79	8.27	5.6
Difference		1.18		2.58		2.2		2	
p		0.18		**0.0047		**0.0050		*0.011	

Physical environment: childhood home overcrowding

Independent sample t-tests were run between individuals with a childhood overcrowding ratio of less than 1 and those at or above 1, in which the overcrowding ratio was defined as the number of individuals living in a home divided by the number of available bedrooms. Individuals were counted as “living” in a home if they slept there for four or more days per week. Bedrooms were defined as closed spaces where individuals sleep for four or more days per week. As seen in Table 2, an overcrowding ratio of less than one (n = 10) was associated with significantly higher depression and anxiety in the early and late pandemic when compared to individuals living with a ratio at or greater than one (n = 199). Results indicate a significant difference in both anxiety and depression during both pandemic stages. Individuals in the lowest childhood overcrowding group (i.e., more individual space) reported higher depression in both the early (p = 0.02*) and late (p = 0.0084**) pandemic; the trend is similar for early- (p = 0.016*) and late- (p = 0.0037**) pandemic anxiety. All mean scores are presented in Table 2.

**Table 2:** Overcrowding ratio relations to pandemic anxiety and depression. Mean depression and anxiety scores of participants by childhood home overcrowding. Data shown indicate that individuals growing up in a home with an overcrowding ratio (individuals living in the home divided by the number of bedrooms) of less than one (reported significantly higher levels of depression and anxiety compared to those living in homes with an overcrowding ratio of greater than or equal to one. Individuals were defined as living in a home if they were present for four or more days of the week, and bedrooms were defined as rooms where individuals slept for four or more nights of the week. p < 0.05*, p < 0.01**

Overcrowding	n	Early Depression	σ	Late Depression	σ	Early Anxiety	σ	Late Anxiety	σ
Ratio < 1	10	16.60	3.13	16.60	3.50	12.80	3.05	13.00	3.20
Ratio ≥ 1	199	13.88	6.20	12.67	6.51	9.87	5.56	9.14	5.51
Difference		2.72		3.93		2.93		3.86	
p		*0.026		**0.0064		*0.016		**0.004	

Physical environment: childhood access to heating and hot water

An independent sample t-test comparison between mean early/late depression and anxiety scores was made for individuals having had access to heating and hot water. While we observed slightly lower CESD-10 and GAD-7 scores in individuals with access to heating versus no access to heating, it was statistically not significant (Appendix B, Table 7). We conducted another independent sample t-test comparison between mean early and late depression and anxiety scores for individuals having had access to hot water while growing up. Although individuals who had access to hot water at eight years of age showed a higher average score for the CESD-10 and GAD-7 at the end of the pandemic in all contexts, differences were not statistically significant (Appendix B, Table 7).

Demographics: political views

Our data indicate that average levels of anxiety and depression were higher for individuals with strong political views, when compared to individuals with moderate political views (Appendix B, Table 9). First, we formed two groups, one consisting of individuals with strong viewpoints, both liberal and conservative, and another with individuals having only slightly polarized or neutral viewpoints (labeled “moderate”). Comparisons suggest that individuals with strong political views were significantly more anxious than those with moderate views by 16.6% in the early (p = 0.0068**) and by 14.3% in the late (p = 0.047*) pandemic. Similarly, higher depression was observed for those with strong political views, although the results were not significant at the 0.05 level for either the early (p = 0.057) or late (p = 0.088) pandemic (Table 3).

**Table 3:** Political view strength relations to pandemic anxiety and depression. Results indicate that participants who held strong political views, whether conservative and liberal, are associated with higher levels of anxiety and depression in the early and late pandemic when compared with individuals with moderate political views. p < 0.05*, p < 0.01**

Views	n	Early Depression	σ	Late Depression	σ	Early Anxiety	σ	Late Anxiety
Strong	95	14.99	6.38	13.82	6.75	11.24	5.54	10.27
Moderate	117	13.37	5.82	12.28	6.20	9.18	5.36	8.75
Difference		1.62		1.54		2.06		1.52
p		0.057		0.088		**0.0068		*0.047

Demographics: age group

We observed no significant trends between age groups after running linear regressions and a correlation in the early and late pandemic, although depression and anxiety appear to decrease slightly as age increases indicating that a larger sample size may have been beneficial (Appendix B, Table 4).

Demographics: biologically assigned birth sex

An independent sample t-test comparison was conducted to compare early- and late-pandemic mean depression and anxiety scores based on sex. Results suggest that there are no differences in mean anxiety or depression symptoms based on sex (Appendix B, Table 8). However, results indicate that females exhibit a consistently higher standard deviation for both the CESD-10 and GAD-7 scores.

Additional analyses

**Figure 4:**
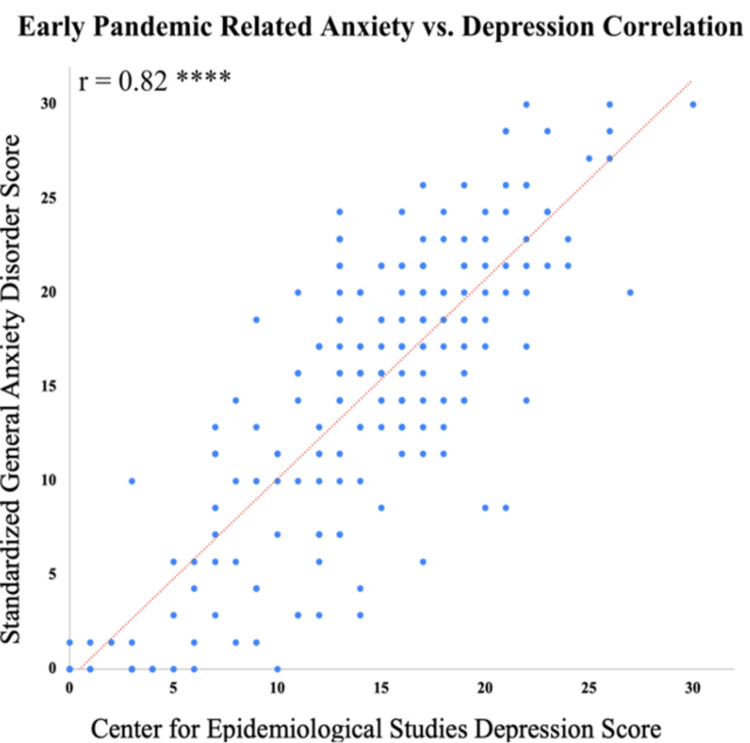
Early-pandemic-related anxiety and depression correlation. During both stages of the pandemic, scores for the GAD-7 and CESD-10 show a strong positive correlation; p (both) = 2.2e-14. GAD-7 scores were standardized to a 30-point scale utilizing Equation 1 (Appendix C). p < 0.05*, p < 0.01**, p < 0.001***, p < 1E-6 ****

**Figure 5:**
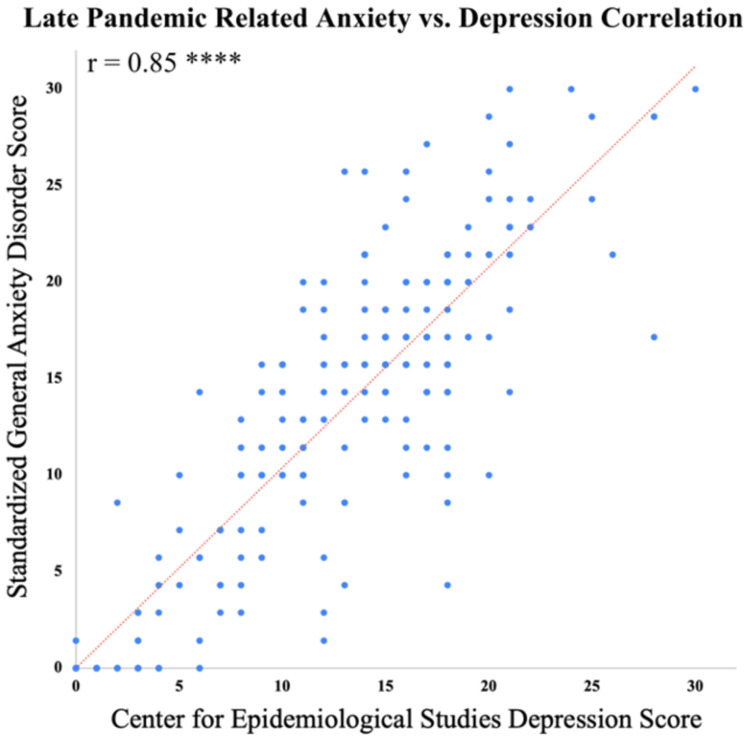
Late-pandemic-related anxiety and depression correlation. p < 0.05*, p < 0.01**, p < 0.001***, p < 1E-6 ****

**Figure 6:**
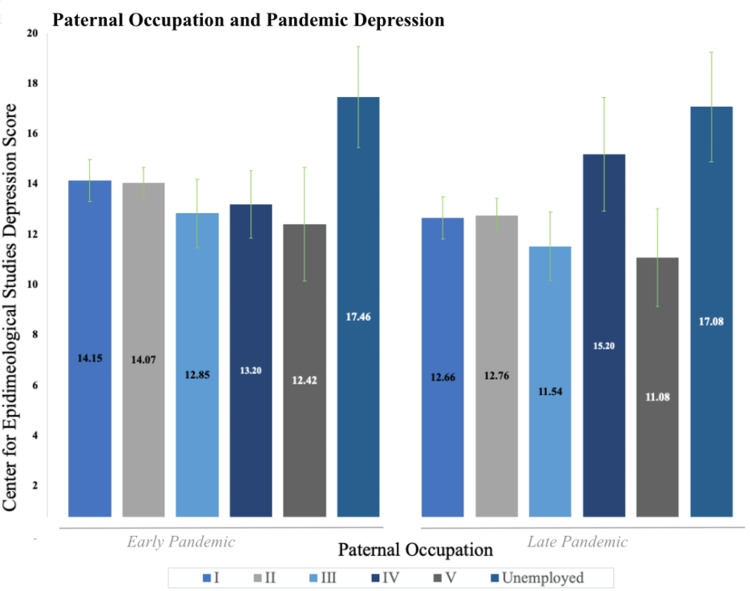
Paternal occupation and pandemic depression. Mean CESD-10 scores by Registrar General’s social class categories based on occupation. Green vertical lines on bars represent the standard error. A similar trend is observed in comparison to paternal anxiety (see Figure 1), but the ANOVA results do not indicate significant differences ((see Appendix B, Table 11).

Additional statistical analyses and results are provided in the appendices. This includes correlations (Appendix A, Figures 4-5; Appendix B, Table 4), non-significant results (Appendix A, Figure 5; Appendix B, Tables 5-8), anxiety and depression scores for more specific political groupings (Table 9), occupations within each classification (Appendix B, Table 10), all ANOVA results (Appendix B, Tables 11-13), 95% confidence intervals for calculated means within significant categorical relationships (Appendix B, Table 14), and effect sizes (Appendix B, Table 15).

## Discussion

Contextualizing results

In the current study, we examined the extent to which childhood socioeconomic factors and demographics are associated with anxiety and depression during the early and late stages of the COVID-19 pandemic. With a variety of heterogeneous associations related to pandemic anxiety and depression symptom severity across childhood SES and demographic variables, it is essential to assess possible mechanisms that may drive these observed differences.

Depression and anxiety for the early and late pandemic, as observed in our data, suggest that there was an overall 7.9% perceived decrease in depression symptoms over the course of the pandemic and an overall 6.6% perceived decrease in anxiety symptoms. While a variety of attributions may explain this, it has been recently found that, in children, the availability of COVID-19 vaccines is related to a decrease in depression symptoms [32]. Therefore, a possible mechanism to explain why participants discerned early pandemic anxiety and depression symptoms as more severe may relate to the availability of vaccines, which were not available during April 2020 but had been available for 22 months by February 2022. Because 91% of our sample had been vaccinated against COVID-19 by February of 2022, it is possible that the observed decrease in psychopathological symptoms over the preceding 22 months may relate to the administration and reception of vaccines. This is further supported by evidence that reception of a COVID-19 vaccine between December 2020 and March 2021 was related to decreased mental distress [47]. Another possibility, although it may not be exclusively separate from vaccination availability, is habituation to stressors over the course of several pandemic years. Other studies have documented the implementation of coping strategies by individuals in both the home and the workplace, which lends support to this explanation, considering that most individuals were in lockdown during April 2020 [30,31].

Using the Registrar General’s social classifications, we grouped individuals into classes I-V based on parental employment status and created a sixth group for unemployed individuals (Appendix B, Table 10). Individuals whose father’s occupation belonged to social class III at eight years of age showed significantly lower levels of anxiety in comparison to the other social classifications, whereas individuals with unemployed fathers in childhood had significantly more anxiety (Figure 1). Interestingly, jobs in our sample from social class III include skilled manual and non-manual occupations; this classification included, but was not limited, to police officers, military personnel, and firefighters. Many of these positions have been long considered “essential,” a concept that was frequently used to describe workers whose jobs required them to report to work during the pandemic; it has also been hypothesized that public sector workers may have experienced increased job security relative to other occupations [48-50]. Because these results allude to paternal occupation, they suggest that early job security from one’s father is related to decreased levels of pandemic-related anxiety, which lends support to the benefits of using a life-course perspective in analyzing psychopathological onset.

Results from our study also indicate a converse relationship; that is, individuals whose fathers had the highest possible job insecurity (unemployment) when they were growing up, reported high levels of anxiety during both the early and late pandemic (Figure 1). This dynamic between paternal occupation, job security, and level of anxiety indicates the importance of growing up in a household with stable parental occupation and further suggests the usefulness of the life-course approach to psychopathology, in which the onset may be significantly influenced by early experiences related to the paternal profession.

In examining the highest household education (of either parent) when an individual was eight years old, we observe a complex dynamic. While the lowest education group (high school) is associated with higher anxiety and depression when compared to trade school/associate degrees, higher education groups (bachelor's and post-baccalaureate degrees) are associated with increased anxiety as well (Figures 2-3). The transition from high school degrees to a trade school/associate degree that marks a significant drop in anxiety and depression may again reflect, to a large extent, the concept of “essential” work. Several “essential” and public-sector occupations during the pandemic, including police officers, plumbers, and more, fall into post-secondary but pre-baccalaureate education levels, again suggesting that early job security of one’s guardians may mitigate later psychopathological symptoms during the pandemic. Our data, therefore, continue to advocate for the idea that higher guardian job security in childhood may relate to decreased perceived levels of anxiety and depression symptoms during the pandemic, implicating a life-course effect of the childhood environment on adulthood mental health afflictions.

We also found that individuals who grew up in highly educated households, such as those with parents holding a bachelor’s or post-baccalaureate degree (master’s and doctoral), indicated higher levels of anxiety on average (Figure 2) compared to individuals with parents holding a trade school/associate degree. Past studies have documented feelings of stress and pressure among children from parents in highly educated households [51,52]. For example, Centers for Disease Control and Prevention (CDC) data indicate that, in the highly educated city of Palo Alto, CA, the youth suicide rate of 14.1 per 100,000 is roughly three times the average in Santa Clara County [53]. The rate in Palo Alto is among the highest in the United States, which has been attributed to familial pressure and academic stress [53,54]. Therefore, it is possible that individuals coming from highly educated households in childhood may be predisposed to higher levels of pressure and stress in the quarantine environment, which could in turn relate to a sense of frustration and a higher onset of anxiety and depression during the COVID-19 pandemic. As a result, our data support the interpretation that individuals with highly educated parents may have experienced increased depression and anxiety resulting from an inability to reach their goals under the imposed COVID-19 restrictions.

Similarly, restrictions imposed by COVID-19 resulted in significant physical environment changes for several careers, for example working from home. A study by Kumar et al. found that in professional careers, ones often requiring a baccalaureate or post-baccalaureate education, there was significant occupational discomfort-related distress during the pandemic [55]. Because higher parental education is related to the higher education of their children [56], it is, therefore, possible that individuals whose parents had higher education levels experienced an increase in depression and anxiety symptoms imposed by restrictions on their personal occupation. This is further supported by Wandberg et al., who found that a higher current education level, as a proxy of concurrent SES, was associated with an increase in depressive symptoms amid the COVID-19 pandemic [57]. This lends support to our notion that parental education may be a reasonable predictor of current education levels of individuals and associated occupations, thus suggesting a possible mechanism for the observed trends in anxiety as a function of the highest household education. Paired with this background, our findings regarding childhood household education indicate a complex, dualistic dynamic such that post-secondary (beyond high school but prior to a bachelor’s degree) education is associated with less pandemic anxiety and depression severity, whereas high (baccalaureate and post-baccalaureate) household education may have the inverse effect.

Assessing the physical environment, it has been put forward that environments under conditions of quarantine correlate with an increase in anxiety and depression [58,59]. However, individuals who grew up in more crowded environments may have been exposed to similar situations in childhood. Studies have found that perceived stress may decrease with higher distress tolerance or increased mindfulness, which is more likely to occur with early exposure to and awareness of a similar stressor [60,61]. Because stress and states of distress have been associated with anxiety and depression during the pandemic, it is possible that childhood exposure to higher home overcrowding and de-individualized space may have prepared individuals for stress under quarantine, hence the association between higher childhood overcrowding and lower levels of anxiety and depression in our findings (see Table 2) [24,62].

It is also important to consider the current employment status as a proxy for current SES. During the pandemic, rates of unemployment jumped as a function of strict global lockdowns, which have been shown to be associated with psychological distress [63,64]. Building on this finding, we examined the relationship between anxiety, depression, and unemployment due to the pandemic, including those who considered themselves to be at risk for unemployment because of the pandemic. Individuals were also able to select that they had no intent to find work, serving as a control. The results indicated that those who are unemployed because of the pandemic or consider themselves to be at risk for unemployment due to the pandemic were associated with significantly higher anxiety in the early and late pandemic and depression in the late pandemic (Table 2).

Job loss has been shown to relate to the onset of anxiety and depression; however, this is not always immediate [65-67]. Past studies have found that long-term or chronic exposure to pandemic-related stressors such as virtual schooling may relate to feelings of learned helplessness in both students and educators [68,69]. It has also been found that extended unemployment may play a role in the onset of both depression and learned helplessness, such as giving up on finding work [7]. Considering that our results indicate that depression was only significantly higher for unemployed individuals in the late pandemic, extended unemployment (or risk for unemployment) may be also associated with learned helplessness [70,71]. This, in turn, may help explain our finding that, between April 2020 and February 2022, symptoms of depression decreased to 14.7% for employed individuals, but only 4% for individuals who were unemployed or at risk for unemployment due to the pandemic (see Results, section 3.6). As these results (job security, and current employment as previously discussed) appear to coincide with results based on childhood SES, there may be merit in examining whether childhood or adulthood sociodemographic environment has a more significant effect on mental health in future studies.

Looking at CESD-10 and GAD-7 scores across the political spectrum, participants in our sample tended to show higher levels of anxiety and depression if they had strong political views, including both liberal and conservative (Appendix B, Table 9). Because of this, an analysis was done by grouping individuals by strength of view, not political ideology. Results indicate that individuals with strong political views had significantly more anxiety; while depression followed the same trend, it slightly exceeded our significance level (Table 3). A possible explanation for this result is to examine the political status of the pandemic, which has been previously theorized to be a cultivator of mass anxiety [72]. Indeed, studies have indicated that the pandemic was associated with increased political polarization, particularly in the United States, with a particularly hot topic being the wearing of masks and vaccination [73,74]. It is therefore plausible that individuals who held strong political views in the pandemic were more affected by highly politicized pandemic reports. Future studies examining the congruence between political ideology and mental health during highly politicized public health events will be useful in understanding what predisposes individuals to anxiety and depression.

Past studies have consistently suggested a general increased psychopathology prevalence among females, as opposed to males. This prevalence has apparently persisted during the pandemic as well: older female adults have been found to exhibit increased levels of anxiety [75,76]. In contrast, we observed no significant difference in the average anxiety or depression scores among males and females in either the early or late pandemic, which may indicate a more universal, non-sex-specific onset of anxiety and depression symptoms amid the pandemic. However, we also found higher standard deviations in scores of female individuals, which indicates that there is a broader distribution in reporting more severe levels of anxiety and depression (Appendix B, Table 8).

Limitations

Our participant sample obtained from the Amazon Mechanical Turk website was predominantly White (85.7%), and thus our findings may not be generalizable to all racial groups. As noted earlier, past research has indicated that, during the pandemic, people of color may have experienced the most significant adverse health outcomes, including those related to mental health. Therefore, it may be beneficial for future studies to specifically recruit participants from various populations to increase the external validity of results. Similarly, in terms of demographics, we report a wide age range between 18 and 76 years. Because a significant portion of our study assesses childhood SES, it is important to recognize that individuals in this study may have grown up at different times surrounded by different social contexts, which may partly impact results. However, because comparisons were standardized to specific variables at eight years of age consistently, any significant skew from societal socioeconomic situations (i.e., recessions) should be limited. Furthermore, in recruiting participants with Amazon Mechanical Turk, we did not target a geographic region, and our survey was available worldwide. However, to maximize the number of participants from the Western hemisphere, we posted small batches of the survey exclusively during daylight hours in the Eastern Standard Time zone of North America. Because participants were not put under exclusionary criteria for pre-existing anxiety and depression due to field bias (see Methods), a slight skew may be present in some findings should certain individuals have had higher symptoms prior to the beginning of the COVID-19 pandemic.

Finally, our study was entirely retrospective, even though questions were asked in the context of both April 2020 (an earlier stage in the pandemic) and the current context (February 2022). Because these data examine retrospective, self-reported symptoms for the early-pandemic time point, results may be partially confounded by biases, including memory limitations and overreporting, and should not be interpreted as longitudinal data. Rather, these data should be interpreted as current as opposed to longitudinal (i.e., how an individual currently views their preceding mental health experiences in 2020, not the severity of depression and anxiety during this time point itself). However, it is important to note that several studies have documented that retrospective measurement is a valid way to investigate earlier experiences in a variety of contexts, particularly when environmental or societal situations cannot be recreated such as the COVID-19 pandemic [77-80]. There is also abundant research that indicates early adverse experiences can impact later mental health, including amid the pandemic, and therefore investigations on how individuals perceive their own early-pandemic mental health are similarly important to examine [37-39]. Chiefly related to our presented analyses, any chance of skewing resulting from recall-based limitations of retrospective answers for the CESD-10 and GAD-7 are equal for all survey participants, and all included participants were required to answer each socioeconomic question. Thus, while this limitation may affect, on some level, comparisons between early and late pandemic cases, it is controlled for comparisons among SES groups in the early pandemic allowing for less biased comparisons.

## Conclusions

Our work suggests that the relationship between parental socioeconomic class in childhood and psychopathological onset in adulthood is complex. Some factors related to childhood socioeconomic class, such as paternal occupation at age eight, suggest that lower childhood SES is associated with poorer mental health during the pandemic. In examining current pandemic-related unemployment, which is often associated with childhood SES, we also found that lower SES individuals were more likely to show poor mental health outcomes amid the pandemic. Other factors related to childhood SES showed an inverse trend. Home overcrowding, for example, another measure of childhood SES, indicated that higher SES individuals may in fact have worse mental health outcomes, particularly if their physical environment in childhood was unreflective of pandemic-related circumstances, such as quarantining or living in close connection with others for extended periods of time. Finally, other factors, such as the highest educational level of parents in a household at age eight, showed a dualistic (U-shaped) relationship with SES and may reflect a complex mechanism. Mental health is affected negatively by job security (having a trade school/associate degree) but is positively affected by the stress from childhood expectations of parents with graduate degrees, and related professional occupations that often require baccalaureate or post-baccalaureate degrees. Importantly, even though memory of anxiety and depressive symptoms has been shown to be reduced as a mental health coping strategy, our results indicate that participants can recall the experience of anxiety and depression in the early pandemic relative to the late pandemic evidenced by higher reports of depression and anxiety symptoms in the early pandemic. Therefore, stressful societal situations may facilitate the long-term retention of these adverse experiences.

Political views of those in our sample might be expected to have a weak, if any, association with childhood SES. Our data suggest that stronger political views of study participants, regardless of whether liberal or conservative, strongly relate to higher levels of pandemic anxiety and depression. This finding suggests that the politicization of widespread social stressors, such as a pandemic, should be closely monitored for potential mental health effects at a societal level. While varied associations between childhood SES and the onset of depression and anxiety are evident in our sample, the mechanisms of occurrence remain unknown. Future studies of psychopathological symptom onset should examine the relationship between current socioeconomic circumstances and childhood parental SES, as indicated by all relevant factors. Our results indicate a complex relationship between childhood SES and pandemic-related anxiety and depression symptoms, in addition to indicating various ties between current demographics and pandemic-related adverse mental health symptoms. Taken together, these associations between early childhood factors and mental health illustrate the importance of applying a life-course perspective in assessing the onset of depression and anxiety. These findings further suggest the importance of understanding the full extent of the childhood environment and how it can influence personal health amid societal stressors, such as a global pandemic.

## Appendices

Appendix A

Appendix B 

**Table 4:** Varied correlations between SES or demographics and psychopathology. Correlations between the CESD-10 or GAD-7 scores with the indicated variables in the early and late pandemic. No correlations were observed between these variables; however, a positive correlational trend presents for paternal occupation, and a negative correlational trend presents for age. A lack of significance may be a result of complicated relationships within certain variables, for example, the heterogeneous associations in education (see Figures 2-3). n.s.: Not significant

	n	r, Early Depression	r, Late Depression	r, Early Anxiety	r, Late Anxiety
Paternal Occupation	205	0.02	0.05	0.07	0.03
Maternal Occupation	209	0.01	-0.003	0.15	0.01
Education	212	-0.08	-0.02	-0.04	0.03
Crowding	210	-0.06	-0.004	-0.08	0.03
Politics	212	-0.03	0.02	0.03	0.07
Age	212	-0.05	-0.02	-0.09	-0.07
p		n.s.	n.s.	n.s.	n.s.

**Table 5:** Mean scores and standard deviations by maternal occupations. Data on maternal occupation suggest no significant differences in mean psychopathology scores between participants with mothers coming from varied socioeconomic classes by occupation. As in Figure 5, occupational class is based on the Registrar General’s social class by occupation, with the addition of an “unemployed” category. n.s.: Not significant

Social Class	n	Early Depression	σ	Late Depression	σ	Early Anxiety	σ	Late Anxiety	σ
I	29	13.55	6.1	10.83	6.59	10.1	5.09	8.17	6.17
II	38	13.11	4.76	13.74	6.71	9.74	5.41	10.21	5.03
III	21	14.57	7.42	12.19	7.73	8.76	6.55	7.62	6.47
IV	4	19.75	7.14	19.75	8.58	13.5	5.45	11.75	7.72
V	5	14.4	8.5	12.2	6.83	7	8.72	6	5.52
Unemployed	91	13.64	6.5	12.37	6.19	9.82	5.62	9.19	5.39
ANOVA, p		n.s.		n.s.		n.s.		n.s.	

**Table 6:** Mean scores for pandemic psychopathology by childhood hot water access. Results indicate that mean psychopathology scores did not significantly differ between participants in the early nor late pandemic. n.s.: Not significant

	n	Early Depression	σ	Late Depression	σ	Early Anxiety	σ	Late Anxiety	σ
Hot Water	162	14.44	6.35	13.10	6.69	10.23	5.81	9.35	5.77
No Hot Water	32	13.41	4.74	12.59	5.93	9.69	4.41	10.06	5.00
p		n.s.		n.s.		n.s.		n.s.	

**Table 7:** Mean scores for pandemic psychopathology by childhood heating access. No significant associations were observed between access to consistent or inconsistent heating and psychopathology scores in the early or late pandemic. n.s.: Not significant

	n	Early Depression	σ	Late Depression	σ	Early Anxiety	σ	Late Anxiety	σ
Consistent Heating	143	14.24	5.83	13.11	6.41	10.24	5.33	9.52	5.47
Inconsistent Heating	14	15.00	5.29	15.86	5.42	11.14	4.88	11.50	4.97
p		n.s.		n.s.		n.s.		n.s.	

**Table 8:** Mean scores for pandemic psychopathology by biologically assigned birth sex. Results indicate that there are no significant differences between psychopathology scores in the early nor late pandemic when comparing biologically assigned birth sex. However, we do note that the standard deviation is consistently higher for female participants, suggesting a higher diversity in psychopathology scores among female participants. n.s.: Not significant

Birth Sex	n	Early Depression	σ	Late Depression	σ	Early Anxiety	σ	Late Anxiety	σ
Male	105	14.61	5.7	13.34	5.95	10.74	5.23	9.86	5.46
Female	107	13.59	6.5	12.61	6.97	12.14	5.76	9.02	5.61
p		n.s.		n.s		n.s		n.s	

**Table 9:** Mean scores for pandemic psychopathology by political views. As noted in Table 3, political views were shown to significantly relate to psychopathology scores on the basis of view strength as opposed to type of view, particularly in terms of higher anxiety. While the trend is less significant, this holds true for depression, particularly for liberal individuals in the early pandemic. p < 0.05* n.s.: Not significant

Views	n	Early Depression	σ	Late Depression	σ	Early Anxiety	σ	Late Anxiety	σ
Strong Liberal (SL)	50	15.62	6.67	13.48	6.95	11.46	5.88	9.88	5.71
Moderate Liberal (ML)	57	13.04	5.92	12.47	5.71	8.75	5.07	8.58	4.73
Moderate (MO)	33	12.97	5.95	12.39	6.51	8.82	5.63	8.67	5.69
Moderate Conservative (MC)	27	14.56	5.49	11.74	6.95	10.52	5.60	9.22	6.46
Strong Conservative (SC)	45	14.29	6.05	14.20	6.57	11.00	5.20	10.71	5.54
p		*ML:SL		n.s.		*ML:SL, *MO:SL, *ML:SC		*SC:SL	

**Table 10:** Jobs within each occupational classification. Participant’s job classifications using Registrar General’s social class categories. Occupations included in the table consist of maternal and paternal occupations.

Class I Jobs	Class II Jobs	Class III Jobs	Class IV Jobs	Class V Jobs	Non-income
Finance	Manager	Police Officer	Security Guard	Factory Worker	Unemployed
Accounting	Administrator	Military	Machinist	Cashier	Housework
Insurance	Marketing	Secretary	Landscaper	Housekeeper	
Engineer	Educator/Teacher	Clerk	Carpet Installer	Newspaper Delivery	
Property Acquisition	Data Analyst	Receptionist	Driver	Excavator	
IT	Auditor	Interior Designer	Sales Workers	Vendor	
Chemist	Chief of Police	Farmer	Substitute Teacher	Janitor	
Healthcare Professionals	CNA, Surgical Assistant	Construction		Refinery Worker	
Wildlife Biologist		Mechanic		Walmart Employee	
Social Worker		Forester		Seamstress	
		Chef		Supermarket Worker	
		Electrician			
		Plumber			
		Fireman			
		Musician/Dancer			
		Hair Stylist			
		Repair Personnel			

**Table 11:** Mean psychopathology comparison by paternal occupation ANOVA. One-way ANOVA results for the average participant depression and anxiety scores in the early and late pandemic grouped by paternal occupation at eight years of age. Results indicate the presence of significant differences among grouping averages for both anxiety conditions. p < 0.05*, p < 0.01**

Paternal Occupation ANOVA	p
Early Pandemic Depression	0.30
Late Pandemic Depression	0.12
Early Pandemic Anxiety	0.016*
Late Pandemic Anxiety	0.005**

**Table 12:** Mean psychopathology comparison by maternal occupation ANOVA. One-way ANOVA results for the average participant depression and anxiety scores in the early and late pandemic grouped by maternal occupation at eight years of age. Results indicate that there are no significant differences among grouping averages for neither depression nor anxiety.

Maternal Occupation ANOVA	p
Early Pandemic Depression	0.48
Late Pandemic Depression	0.16
Early Pandemic Anxiety	0.60
Late Pandemic Anxiety	0.30

**Table 13:** Mean psychopathology comparison by the highest household education ANOVA. One-way ANOVA results for the average participant depression and anxiety scores in the early and late pandemic grouped by highest household education at eight years of age. Results indicate the presence of significant differences among grouping averages for both anxiety conditions, in addition to depression in the early pandemic.

Education ANOVA	p
Early Pandemic Depression	0.037*
Late Pandemic Depression	0.20
Early Pandemic Anxiety	0.018*
Late Pandemic Anxiety	0.029*

**Table 14:** Confidence intervals for psychopathology score means. 95% confidence intervals for all mean depression (CESD-10) and anxiety (GAD-7) scores presented in the manuscript text.

Variable	Group	Condition	95% Confidence Interval
Paternal Occupation	Class I	Early Depression	14.08 +/- 1.59
		Late Depression	12.66 +/- 1.6
		Early Anxiety	9.85 +/- 1.52
		Late Anxiety	9.25 +/- 1.59
	Class II	Early Depression	14.07 +/- 1.17
		Late Depression	12.76 +/- 1.31
		Early Anxiety	10.3 +/- 1.06
		Late Anxiety	9.72 +/- 1.05
	Class III	Early Depression	12.85 +/- 2.66
		Late Depression	11.54 +/- 2.68
		Early Anxiety	7.85 +/- 2.08
		Late Anxiety	6.38 +/- 1.86
	Class IV	Early Depression	13.2 +/- 2.64
		Late Depression	15.2 +/- 4.44
		Early Anxiety	10.6 +/- 2.91
		Late Anxiety	11.5 +/- 3.59
	Class V	Early Depression	12.42 +/- 4.45
		Late Depression	11.08 +/- 3.81
		Early Anxiety	7.75 +/- 3.62
		Late Anxiety	7.08 +/- 3.18
	Unemployed	Early Depression	17.14 +/- 3.92
		Late Depression	16.71 +/- 4.27
		Early Anxiety	13.93 +/- 3.03
		Late Anxiety	12.57 +/- 2.95
Household Education	High School	Early Depression	16.78 +/- 2.08
		Late Depression	13.7 +/- 2.06
		Early Anxiety	11.35 +/- 1.88
		Late Anxiety	9.39 +/- 1.9
	Post-Secondary	Early Depression	11 +/- 2.48
		Late Depression	9.93 +/- 2.03
		Early Anxiety	5.93 +/- 1.59
		Late Anxiety	5.4 +/- 1.75
	Bachelor's	Early Depression	13.89 +/- 1.08
		Late Depression	13.38 +/- 1.15
		Early Anxiety	10.24 +/- 0.97
		Late Anxiety	9.87 +/- 0.94
	Post-Baccalaureate	Early Depression	14.2 +/- 1.82
		Late Depression	12.2 +/- 2.05
		Early Anxiety	10.24 +/- 1.75
		Late Anxiety	9.24 +/- 1.82
Unemployment	Employed	Early Depression	14.76 +/- 1.12
		Late Depression	14.17 +/- 1.17
		Early Anxiety	11.07 +/- 0.97
		Late Anxiety	10.27 +/- 1.024
	Unemployed/Risk	Early Depression	13.58 +/- 1.32
		Late Depression	11.59 +/- 1.13
		Early Anxiety	8.87 +/- 1.16
		Late Anxiety	8.27 +/- 1.13
Overcrowding	Ratio < 1	Early Depression	16.6 +/- 1.94
		Late Depression	16.6 +/- 2.17
		Early Anxiety	12.8 +/- 1.89
		Late Anxiety	13 +/- 1.98
	Ratio ≥ 1	Early Depression	13.88 +/- 0.86
		Late Depression	12.67 +/- 0.77
		Early Anxiety	9.87 +/- 0.77
		Late Anxiety	9.14 +/- 0.77
Politics	Strong Views	Early Depression	14.99 +/- 1.28
		Late Depression	13.82 +/- 1.36
		Early Anxiety	11.24 +/- 1.11
		Late Anxiety	10.27 +/- 1.13
	Moderate Views	Early Depression	13.37 +/- 1.06
		Late Depression	12.28 +/- 1.12
		Early Anxiety	9.18 +/- 0.97
		Late Anxiety	8.75 +/- 0.98

**Table 15:** Effect sizes for significant relationships. Effect sizes for all significant relationships presented in the manuscript text. Results predominantly indicate a small-to-moderate effect size for significant relationships.

Variable	Condition	r (t-test)	f (ANOVA)
Early vs. Late Pandemic Depression	N/A	0.089	
Early vs. Late Pandemic Anxiety	N/A	0.06	
Paternal Occupation	Early Depression		0.15
	Late Depression		0.19
	Early Anxiety		0.25
	Late Anxiety		0.28
Household Education	Early Depression		0.21
	Late Depression		0.14
	Early Anxiety		0.24
	Late Anxiety		0.23
Unemployment	Early Depression	0.095	
	Late Depression	0.20	
	Early Anxiety	0.20	
	Late Anxiety	0.28	
Overcrowding	Early Depression	0.27	
	Late Depression	0.35	
	Early Anxiety	0.31	
	Late Anxiety	0.39	
Politics	Early Depression	0.13	
	Late Depression	0.12	
	Early Anxiety	0.19	
	Late Anxiety	0.14	

Appendix C

Equation 1

$GAD-7standardized = (30/21) * GAD-7original$

GAD-7 standardization for supplementary anxiety vs. depression correlation plots

This standardization equation was used for the creation of only Figure 4.
